# New Insights and Emerging Therapeutic Approaches in Prostate Cancer

**DOI:** 10.3389/fendo.2022.840787

**Published:** 2022-02-11

**Authors:** Fabrizio Licitra, Pia Giovannelli, Marzia Di Donato, Alessandra Monaco, Giovanni Galasso, Antimo Migliaccio, Gabriella Castoria

**Affiliations:** Department of Precision Medicine, University of Campania ‘L. Vanvitelli’, Naples, Italy

**Keywords:** nerve growth factor signalling, calcium influx, cancer-associated fibroblasts, prostate cancer, new drugs

## Abstract

Prostate cancer is the second most frequently diagnosed cancer in men and several therapeutic approaches are currently available for patient’s care. Although the androgen receptor status represents a good predictor of response to androgen deprivation therapy, prostate cancer frequently becomes resistant to this approach and spreads. The molecular mechanisms that contribute to progression and drug-resistance of this cancer remain still debated. However, few therapeutic options are available for patient’s management, at this stage. Recent years have seen a great expansion of the studies concerning the role of stromal-epithelial interactions and tumor microenvironment in prostate cancer progression. The findings so far collected have provided new insights into diagnostic and clinical management of prostate cancer patients. Further, new fascinating aspects concerning the intersection of the androgen receptor with survival factors as well as calcium channels have been reported in cultured prostate cancer cells and mouse models. The results of these researches have opened the way for a better understanding of the basic mechanisms involved in prostate cancer invasion and drug-resistance. They have also significantly expanded the list of new biomarkers and druggable targets in prostate cancer. The primary aim of this manuscript is to provide an update of these issues, together with their translational aspects. Exploiting the power of novel promising therapeutics would increase the success rate in the diagnostic path and clinical management of patients with advanced disease.

## Introduction

Prostate cancer (PC) still remains the second most commonly diagnosed neoplasia in men (1). Depending on the availability of specific screenings, including the prostate-specific antigen (PSA) assay, the lifestyle and environmental factors, PC incidence varies among men of different ethnicities (2). Despite the recent advances in early diagnosis and detection, the disease’s onset is often asymptomatic, accounting for numerous late diagnoses. Additionally, the prognosis can be favorable at early stage’s disease, given the progresses of advanced radiotherapy technology (3, 4 and refs therein). However, PC still represents the second leading cause of cancer-related death in men, albeit its mortality rate is relatively low (almost 20-30%), as compared with other solid cancers (5). New therapeutic options are needed for patients with advanced disease.

PC pathogenesis and progression depend on androgen/androgen-receptor (AR) circuit. As such, the mainstream pharmacological approach relies on the androgen deprivation therapy (ADT), which shows a satisfactory response in a significant number of cases. However, many PC relapse and progress towards a more aggressive phenotype, often characterized by ADT insensitivity and androgen-independence. Such phenotype, also called castrate-resistant prostate cancer (CRPC), may be metastatic or not (6, 7). Additionally, a subset of PC might further differentiate into neuroendocrine phenotype, also called neuroendocrine PCs (NEPCs). These cancers lose AR signaling, become more aggressive and exhibit androgen-independence in a quite scantly known molecular landscape (8). Among the various factors elsewhere excellently discussed (9–11), PC progression is often characterized by abnormal AR-mediated signaling activation or AR variants (12), which might help the tumor to achieve ADT unresponsiveness. However, emerging findings have identified unexpected drivers of PC progression. Some of them are implied in the survival response elicited by the receptor tyrosine-kinase (RTK) signaling, such as the nerve growth factor (NGF) and its high-affinity receptor, tropomyosin-related kinase receptor A (TrkA; 13). Recent papers, including ours, have investigated this issue in PC (14–18). Other findings have identified the transient receptor potential melastatin-8 (TRPM-8) as a playmaker in PC (19–21 and refs therein), likely because of its role in connecting the androgen endocrine system with intracellular calcium levels (22). At last, the role of cancer-associated fibroblasts (CAFs) in PC progression is undeniable (23). The finding that CAFs harbor significant amounts of AR has opened new ways for a better understanding of the role of tumor microenvironment in PC progression and more tailored approaches of this cancer (23–29).

In search for a link between these three apparently unlinked items, it might be argued that NGF and other neurotrophins secreted from PC cells or CAFs sustain tumor survival and aggressiveness through a paracrine loop, as it occurs in breast cancer (30–33). However, NGF and calcium signaling might intersect each other in PC, as it occurs in neurons (34) or Schwann cells (35). In this manuscript, we will discuss these emerging findings and their connections. The potential application of these data in diagnostic and therapeutic guidance of PC will be outlined.

## Nerve Growth Factor and its Receptors in Prostate Cancer

**Figure 1:**
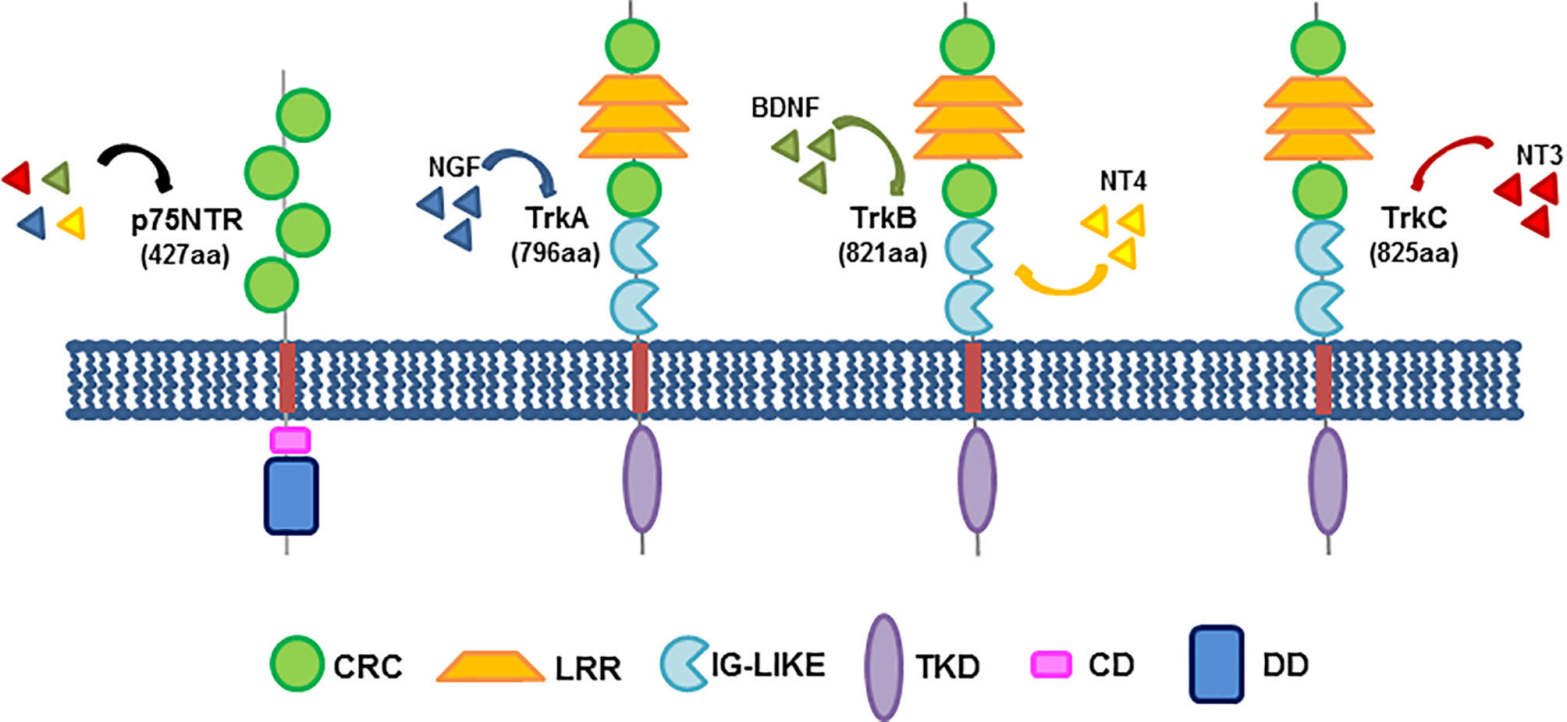
Neurotrophin receptors and their structure. The three members of the Trk family share a common structure, encompassing a cysteine-rich cluster (CRC), three leucine-rich repeats (LRR), a second cysteine cluster and two immunoglobulin-like domains (IG-Like), responsible for ligand binding, in their extracellular domains. A transmembrane domain (TMD) anchors the receptor to the plasma membrane. The intracellular region consists of the tyrosine kinase domain (TKD). On the left, the low-affinity neurotrophin receptor, p75NTR (called NGFR) is also depicted. It consists of four cysteine-rich clusters in the extracellular domain, a transmembrane region and an intracellular region exhibiting a chopper domain (CD) and a death domain (DD). Shown beside each Trk member is reported the corresponding high-affinity binding neurotrophin. p75NTR might bind all neurotrophins at low affinity. The indicated domains are colored as follows: CRC, green; LRR, yellow; IG-Like, light blue; TMD, red; TKD, purple; CD, pink; DD, dark blue.

The action of neurotrophins is mediated by the binding to membrane receptors, mainly the neurotrophin receptor p75NTR (also called NGF receptor; NGFR) and the neurotrophin tyrosine kinase receptor (Trk) family, which consists of three members, TrkA, B and C, with a variable affinity for the four identified neurotrophins (NGF; brain-derived neurotrophic factor, BDNF; neurotrophin 3 and 4, NT3 and NT4; **Figure 1**). Their dependent signaling mediates, indeed, the activation of several downstream effectors, such as MAPK, PI3-K, PLCγ as well as the small GTP binding proteins that strongly impact differentiation and survival, cytoskeletal remodeling, receptor cross-talk and ion channels in various cell types, other than neurons (13). Beyond their role in neuronal cells, neurotrophic factors are emerging as potential drivers of cancer progression, and therapies specifically affecting the neurotrophin-mediated signaling might hold value for innovative treatments of human cancers (36, 37).

PCs release NGF and express the neurotrophin receptors, which undergo significant changes during PC progression, as primary PC express both TrkA and NGFR, while losing NGFR during the disease progression. This behavior has been linked with PC onset and androgen-resistance development (38). At last, NGFR is almost completely absent in metastatic PC, making the TrkA receptor the lead driver of NGF signaling in aggressive PC (16). Previous findings have reported that NGF triggers mitogenesis and promotes PSA release through TrkA activation in LNCaP cells, and this effect is additive to that exerted by androgens (39). As such, it was thereafter found that the tyrosine kinase inhibitor, CEP-701 blocks the TrkA-mediated events, thereby reducing invasiveness of PC cultured cells (40). Derangements of NGF and its dependent signaling can be often detected in PC (41), where the neurotrophin might be released by PC cells and/or the surrounding stromal cells. As such, a paracrine loop between the two counterparts occurs (42). We recently reported that a reciprocal cross-talk between AR and TrkA fosters the mitogenesis or motility of LNCaP cells in response to NGF or androgens (15). The obvious impact of these findings is that combinatorial treatment with antiandrogens and TrkA inhibitors might be explored in PC patients. Consistent with the hypothesis that TrkA is involved in PC motility and spreading, it has been shown that non-proteolytic ubiquitination of TrkA by the ubiquitin-ligase TRAF4 increases the kinase activity of the receptor and mediates PC spreading. The finding that TRAF4 is overexpressed in advanced PC specimens strongly supports the involvement of this mechanism in PC aggressiveness (14). The role of NGF in PC malignancy has been further highlighted by the finding that NGF-elicited activation of TrkA increases mitogenesis, epithelial-mesenchyme transition (EMT) and invasion of various CRPC-derived cells through activation of the downstream Ras- and PI3-K-dependent signaling cascades. Chemical inhibitors of TrkA or siRNA approaches have definitely indicated a role for this receptor in NGF-elicited responses of CRPC-derived cells or spheroids (16). In addition to suggesting the clinical benefit from TrkA inhibitor usage in PC patients (37), the findings so far presented point to the role of NGF axis in PC survival. This circuit might substitute the androgens in controlling PC cell survival and lead to disease’s progression towards the CRPC stage. As before stated, similar findings have been reported in various ‘hormone-dependent’ cancers, including breast cancers. Thus NGF might intersect the steroid endocrine system in various solid cancers.

Beyond the well described mechanism(s) responsible for PC progression and drug-resistance, neuroendocrine differentiation of PC is recently emerging as a process by which a subset of CRPC escapes the ADT. These tumors acquire some signatures (low or absent AR signaling, Rb and p53 loss, amplification of Myc-N and epigenetic changes) which lead to a highly aggressive phenotype and patient’s death within 2 years (8 and therein refs). To date, no therapies are available for NEPC patients. Therefore, the identification of NEPC drivers represents a major challenge. Some years ago, it was shown that PC cells overexpress Myc-N after a prolonged ADT. The oncogene activation correlated with a low or absent AR expression. It was proposed that this feature leads to development of undifferentiated, invasive PC cells that exhibit characteristics similar to those of human NEPC (43). Subsequent reports have confirmed that a small fraction of PCs differentiate into NEPCs upon protracted ADT. These cancers lose the AR-dependent signaling and progress towards an aggressive phenotype, whose molecular drivers are still under investigation (44). Simultaneously, it was shown that ZBTB46 transcription factor might act as one of these key players. It induces NGF expression upon a prolonged ADT treatment in PC patients. Mechanistically, NGF interacts with the peripheral nerve cholinergic receptor muscarinic 4 (CHRM4) to trigger PC cell differentiation by AKT and Myc-N activation. These events might finally lead to the development of neuroendocrine phenotype and ADT-resistance (18). In addition to identifying ZBTB46 as a signature for NEPC, these findings significantly contribute to the understanding of unwanted effects caused by prolonged ADT in PC patients.

Although the role of NGF and its dependent signaling in PC pathogenesis and progression is well established, genetic aberrations of NGF receptors have not been so far reported in PC (45). Thus, derangements of NGF-signaling caused by deregulation of the NGF-RTK or excessive production of NGF might be involved in PC progression. An increased expression and/or release of NGF was firstly detected in human PC specimens and PC-derived cell lines (46) and subsequently confirmed by several labs. Neurotrophic factors can be currently assayed in urine samples from PC patients (47), thus indicating a reliable, non-invasive approach for detection of novel PC biomarkers in body fluids. Extension of these findings to a large cohort of PC patients might expand the current strategies for patient’s stratification. By contrast, RTK derangements cannot be easily detected. Nevertheless, from the findings previously discussed, it appears that some biomarkers, such as TRAF4 or ZBTB46, emerge as predictors of TrkA activation. Their overexpression correlates with aberrant TrkA activation and metastatic events or would predict, as in the case of ZBTB46, the increase in NGF levels with the subsequent signaling derangement in PC patients. These findings, together with the well-established role of neurotrophins for autonomic innervation of PC into the tumor microenvironment, indicate that NGF and their receptors are clinically actionable in PC (48 and therein refs). On the basis of preclinical findings (40, 49), the RTK inhibitor, lestaurtinib (CEP-701) entered clinical trials (NCT00081601) in PC patients, with promising data from phase I studies. However, the drug failed to show a significant PSA response in patients with localized hormone-refractory PC (50). Subsequently, another small-molecule RTK inhibitor, cabozantinib was approved for the treatment of metastatic PC. Noteworthy, these and other currently used inhibitors, such as NCT02219711, block a broad range of RTK and frequently induce side-effects, mainly the drug-resistance. Thus, only in-depth investigation in 3D models from PC specimens or patient’s derived xenografts might allow a more tailored therapy. In this regard, the design and synthesis of small bioavailable peptides specifically perturbing the key signaling functions of NGF receptors can be envisaged. Similar approaches have been successfully applied in our lab to disrupt the upstream interactions of sex steroid receptors with signaling effectors in quite different experimental settings (15, 29, 51–55).

Lastly, it cannot be neglected the role of NGF in PC-related pain, which is the most common symptom of PC bone metastasis (56). Anti-NGF blocking antibodies were firstly used to reduce PC-related bone pain in mouse models. Interestingly, this pharmacologic approach showed limited adverse effects, as compared with nonselective non-steroidal drugs or opioids (57). After many years of investigations, we are now aware that neurotrophins control the autonomic innervation of tumor microenvironment and, hence, PC progression. As such, neurotrophic factor assays would predict the progression and metastatic events in PC patients. Their pharmacologic manipulation can be used to prevent PC progression, reduce the PC-related bone pain and improve the quality of life in patients (48).

## The Role of Transient Receptor Potential Melastatin-8 (TRPM8) in Prostate Cancer

The calcium channels mediate activation of several intracellular pathways by modulating the influx of cations (58 and therein refs). Some of them have emerged in recent years as important players in PC pathogenesis. As such, they represent ‘druggable’ targets in PC therapy (59, 60).

**Figure 2:**
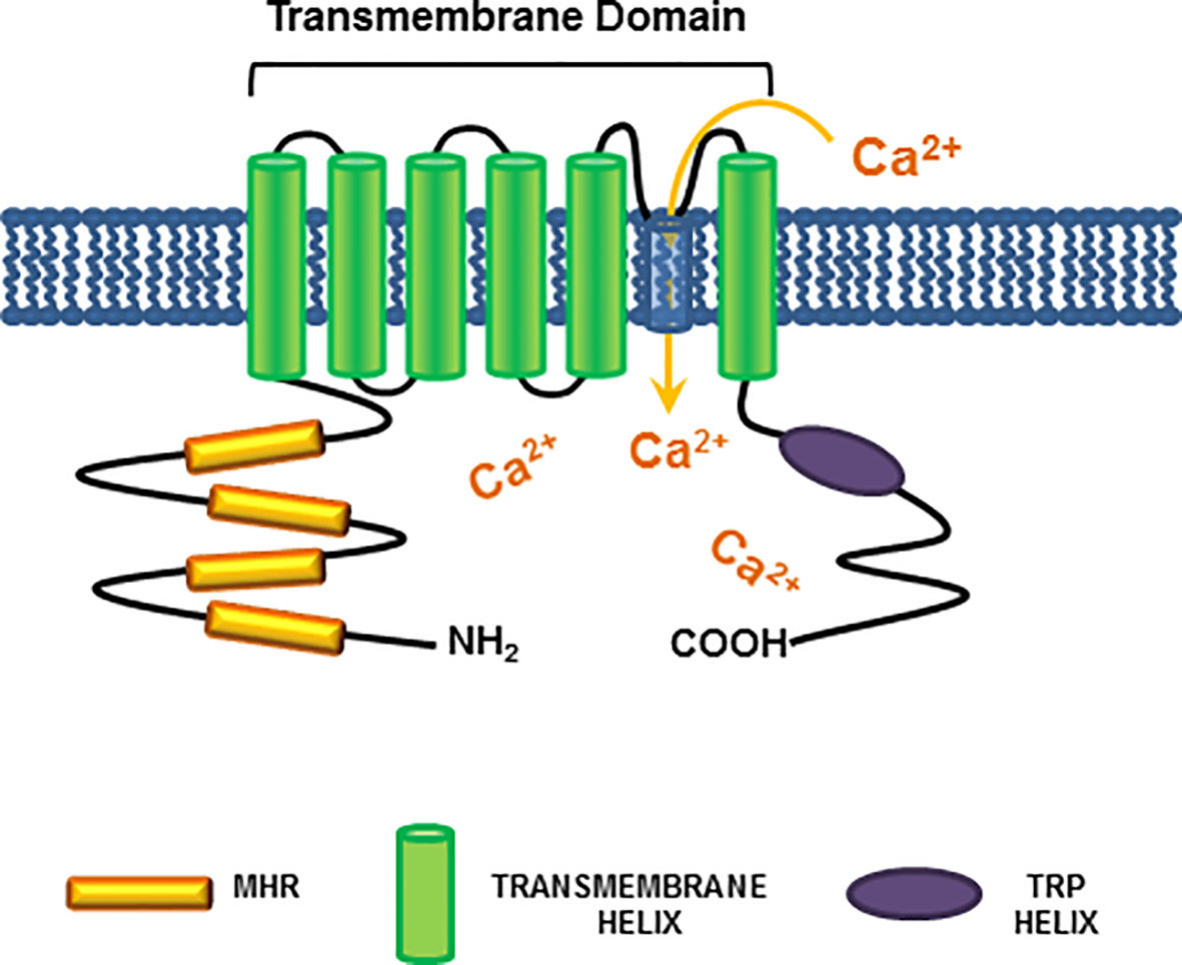
TRPM8 structure. A schematic representation of the TRPM8 channel shows all the common structures between TRPM members. The cytosolic N-terminal region is connected to four Melastatin Homology Regions (MHRs), which are required for channel assembly and ion trafficking. The MHRs are connected to six helices within the plasma membrane, which constitute the trans-membrane domain (TMD). At last, the intracellular TRP helix is connected *via* a coiled-coil domain to the C-terminal domain (CTD). The indicated domains are colored as follows: MHR, orange; TMD helices: green; TRP helix: purple. As indicated in Figure, TRPM8 channel activation leads to an increase in cytosolic calcium levels.

The transient receptor potential melastatin-8 (TRPM8) is a Ca^2+^-permeable cold-sensing channel, which has received increasing attention for its role in a *plethora* of human solid cancers, including PC (20, 61–63). TRPM8 belongs to a family of eight members, classified from TRPM1 to TRPM8. They share a common structure, including a N-terminal region of almost 700 amino-acids, which endows four Melastatin Homology Regions (MHRs). MHRs are required for channel assembly and ion trafficking. The channels also exhibit a trans-membrane domain (TMD) with six helices, together with an additional intracellular helix (TRP helix). The C-terminal coiled-coil domain links the C-terminal domain (CTD) to the TRP helix (64). **Figure 2** illustrates the TRPM8 molecular organization. Among the various members, TRPM8 is involved in ion’s homeostasis and it is sensitive to redox-state and temperature changes. Additionally, the channel can be activated by thermal stimuli (cold), depolarization of cell membranes or chemical compounds, such as the menthol and icilin (65).

TRPM8 expression was initially discovered in sensory neurons (66, 67). Subsequent studies of genome wide expression profiling showed that it is abundantly expressed in prostate tissue and PC samples (68, 69). TRPM8 expression is regulated by androgens, while ADT and the androgen-independence status both reduce its expression in PC tissues (69). Again, negligible levels of TRPM8 can be detected in PC3 and DU145 cells (22, 68, 70). These data highlight the importance of the TRPM8 channel in PC progression and hint at the usage of TRPM8 as a prognostic marker of PC progression.

Low levels of TRPM8 were detected at plasma membrane or endoplasmic reticulum of the androgen-dependent LNCaP cells. In these cells, the channel activation leads to mitogenesis by regulating Ca^2+^ and Na^+^ homeostasis (70). Subsequent studies in the same cells have shown that the *trpm8* gene is responsive to androgens, as its hormone regulation can be mediated by an androgen response element (ARE). Beyond this transcriptional regulation, a ligand-regulated degradation of TRPM8 has been detected in LNCaP cells (71). These findings underline the importance of hormone regulation in TRPM8 expression, at both transcriptional or post-translational level. Such regulation might impact the proliferation, survival and motility of PC cells. However, TRPM8 can also be regulated by PSA. In PC3 cells engineered to overexpress TRPM8, PSA acts as a channel agonist, prompting the Ca^2+^ intake. As such, bradykinin 2 receptor (B2R) activation occurs, with the consequent activation of protein kinase C pathway and inhibition of cell migration (72). Thus, once released, PSA activates TRPM8 by an autocrine loop, thereby impairing the invasive potential of PC cells. These findings point to the protective role for TRPM8 in PC. Recent findings have consistently shown that WS-12, a selective TRPM8 agonist, sensitizes the locally advanced PC to a sublethal dose of X-rays. These findings indicate that pharmacologic manipulation of TRPM8 by agonists would avoid the side effects correlated to high-dose ionizing radiation approach in PC patients (63).

We have recently reported that androgen stimulation of various PC-derived cells rapidly induces the complexation of AR with TRPM8 at extra-nuclear level. Previous findings have consistently shown that androgens trigger the AR/TRPM8 interaction within the lipid rafts microdomains of PC3 cells engineered to express AR (73). Whatever the intracellular localization of the complex, our data indicate that the androgen-induced AR/TRPM8 complex assembly controls the aggressive behaviour of PC cells through the increase in cytosolic [Ca^2+^] levels. Newly synthesized TRPM8 antagonists revert these effects, impair the mitogenesis and invasion of PC cells and reduce the growth of PC cell-derived spheroids. Remarkably, the designed antagonists impair the proliferation and invasion of CRPC cells still expressing AR or the AR-V7 variant (22). As this mutant confers the anti-androgen resistance to PC patients (74), our recent study indicates that TRPM8 channel is clinically actionable in CRPC patients. In summary, we posit that TRPM8 acts as a molecular link between the androgen- and calcium-dependent signaling in PC. Therefore, the discovery of new selective TRPM8 antagonists hold promising results in PC therapy, since the lead compounds we used combine the selective modulation of AR-mediated rapid actions with the release of intracellular calcium. This combinatorial approach may be more effective than the currently used ADT. The arguments put forward here, together with the recent identification of TRPM8 mRNA as a bloodstream signature for PC aggressiveness (75), strongly encourage further studies in this direction.

The finding that TRPM8 regulates key features of PC cells call for additional comments. Other members of the same family can be regulated by sex steroids. TRPM4 and TRPM6, for instance, have been linked to non-transcriptional estrogen action in various cell types (76, 77). Pregnenolone sulfate, the precursor of steroid hormones, transiently activates TRPM3, thereby increasing the calcium influx and the insulin secretion from pancreatic islets (78). As such, the TRPM family members connect the steroid endocrine system with calcium and insulin pathways. Notably, NGF induces through TrkA signaling activation the up-regulation of TRPM8 in neuronal cells. This process requires the reversible activation of the Src tyrosine kinase as well as PI3-K (79), the two mainstream effectors activated by rapid actions mediated by AR in target cells. Thus, it might be argued that an intricate network made up of TrkA/TRPM8/AR components sustains the activation of pathways triggered by NGF or androgens or calcium in PC. If that were to happen, drug escape would easily occur. As such, ADT or TrkA inhibitors or even TRPM8 antagonists might fail in monotherapy.

## The Role of Cancer Associated Fibroblasts in Prostate Cancer Pathogenesis and Progression

CAFs represent the most important component of the tumor microenvironment (TME), which surrounds the neoplastic tissue. Together with the extracellular matrix (ECM), blood vessels and immune cells, CAFs have emerged as key regulators of cancer cell proliferation and metastasis. They respond to the tumor-released growth factors or cytokines and secrete cytokines, chemokines and growth factors of their own. Again, by depositing or degrading ECM proteins, CAFs also influence the TME architecture, thus creating a favorable or a disadvantageous environment for the onset and progression of several tumors. The study of CAFs origin and analysis of their actions have emerged during the last years as a main road for a better understanding of cancer pathogenesis and progression, as well as the design of novel strategies for diagnostic and therapeutic guidance in patients (*reviewed in* 80, 81).

In normal tissues, fibroblasts contribute to the production of ECM and are major players in restoring the tissue integrity upon injury or chronic damage. Under such conditions, they acquire an activated phenotype, characterized by the expression of a subset of mesenchymal markers, including α-smooth muscle actin (α-SMA), fibroblast activating protein (FAP) and vimentin (82, 83). At this stage, they become myofibroblasts, contribute to the tissue recovery by releasing cytokines, chemokines and growth factors, such as the TGF-β and the vascular endothelial growth factor A (VEGF-A), and also recruit immune cells. CAFs derived from neoplastic tissues lack the markers for epithelial/endothelial lineages, show a decrease in CD36 expression and exhibit few genetic mutations, in the presence of an elongated cell morphology. They can be detected before the onset of a proper neoplasia, as a consequence of a tissue injury or damage. However, CAFs may also arise from a population of fibroblasts surrounding primitive lesions, with the initial aim to suppress the tumor growth. Additional events might switch this function, addressing them towards a tumor-supporting activity. Other studies have also indicated a possible, though less likely, origin of CAFs from mesenchymal stem cells, pericytes or adipocytes in different tumor types. Finally, CAF activation typically depends on soluble molecules, including interleukins and growth factors, which activate several intracellular signaling pathways, such as the TGF-β, Notch and NF-κB ones. Direct contact between cancer cells and fibroblasts could be also responsible for their activation, even if only in some cancer types. Simultaneously, other TME components could induce fibroblasts activation, regardless of cancer cells presence (81, 84).

**Figure 3:**
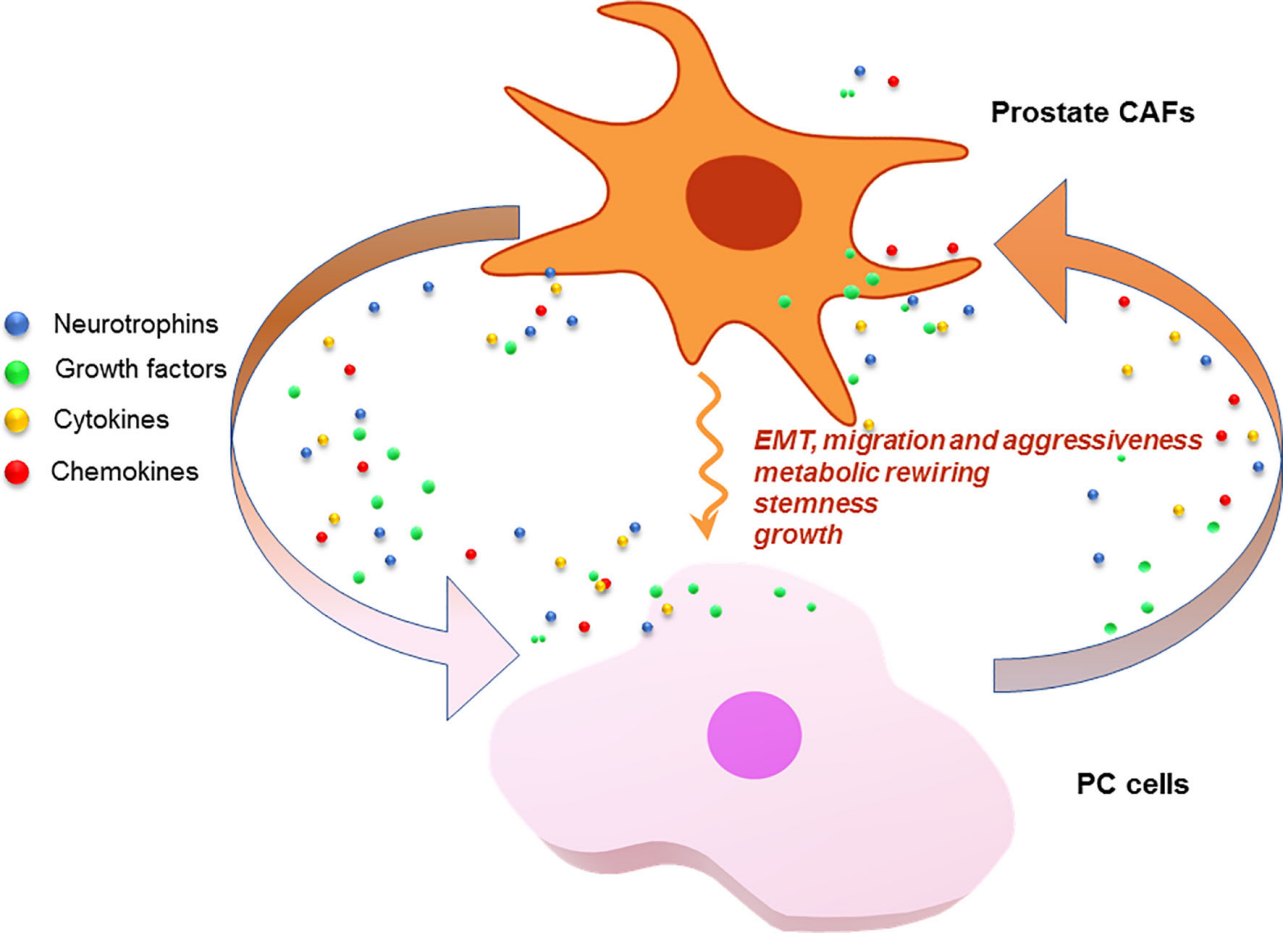
CAFs and PC cells as signal exchangers. CAFs or PC cells release neurotrophins, growth factors, cytokines and chemokines. These signals are exchanged by the cells to foster a *plethora* of responses that lead to PC progression and drug-resistance.

Pioneering studies have pointed to the fundamental role of CAFs in supporting the onset of PC (85). Currently available data indicate that prostate CAFs establish a reciprocal, paracrine interaction with PC cells, resulting in EMT of the latter. Interleukin-6 (IL-6) produced by PC3 cells activates fibroblasts derived from patients with benign prostatic hyperplasia (BPH). Interestingly, different markers of fibroblast activation can be detected in these conditions, as compared with those expressed upon TGF-β-mediated activation (86). At the same time, CAFs activated by IL-6 promote EMT of PC3 cells and increase invasiveness in a similar fashion to that achieved by TGF-β-activated fibroblasts. Interestingly, only transformed prostate cells trigger CAFs activation, and such skill strictly depends on cell aggressiveness. Unlike PC3 or DU145, androgen-sensitive LNCaP cells seem, indeed, unable to activate CAFs. The mechanism by which PC cells acquire invasive phenotype, together with the EMT, depends on secretion of matrix metalloproteinases 2 and 9 (MMP2, MMP9) by CAFs. This response can be only detected on IL-6 stimulation and is reversed by MMPs inhibitors, thus indicating a role for MMPs in the observed findings. Additionally, CAFs are required for PC onset and metastatic spreading in mouse xenograft models, since their absence impairs the PC3 cell ability to generate subcutaneous tumors. Again, CAF-induced EMT of PC cells contributes to the formation of PC stem cells, thus enhancing cancer stemness (87). CAFs also play an energy-supporting role in PC, since prolonged exposure of PC3 and DU145 cells to CAF-conditioned media increases PC cell mitochondrial activity through sirtuin 1/peroxisome proliferator-activated receptor gamma coactivator 1-α (SIRT1/PGC-1α) axis. SIRT1 or PGC-1α silencing impairs the CAF-induced EMT in PC3 cells. Moreover, metabolic deregulation of PC cells by CAFs ultimately increases PC cells invasive potential by stabilization of hypoxia-induced factor 1α (HIF-1α), a reported feature of enhanced PC cell malignancy. Additionally, CAFs are also able to transfer horizontally and unidirectionally their dispensable, functional mitochondria to PC cells, thus contributing to their increased metabolic capacity, both directly or through activation of the SIRT1/PGC-1α pathway. By this mechanism, CAFs establish a symbiotic interaction with PC cells, which sustains their proliferative and metastatic behavior through metabolic regulation, production of high-energy metabolites and mitochondria supply (88). Recent findings have also indicated a role for CAFs in the development of castration-resistance. Accordingly, PC resistance to the 2^nd^ generation antiandrogen, enzalutamide, would depend on CAFs, as shown in a PC mouse model progressing from castration-sensitive to castration-resistant state. The process seems mediated by the activation of the specific NRG1 receptor, HER3, by CAFs-secreted neuregulin-1 (NRG-1). Altogether, the proposed findings identify the NRG1/HER3 axis as a main candidate in the antiandrogen resistance. In support of this concept, high levels of NRG1, together with an enhanced NRG1 mRNA expression, can be detected in CAFs from ADT-treated PC patients (17). These results suggest that targeting of the NRG1/HER3 axis may be beneficial in CRPC patients. **Figure 3** schematically depicts the interplay between CAFs and PC cells.

However, the findings so far presented raise an important question. It concerns the expression of AR in CAFs. Previous findings from prostate CAFs have shown that the receptor is expressed at lower levels, as compared with LNCaP cells. Its somatic knockdown reduced the proliferative and migratory potential of PC3 cells, suggesting a role for stromal AR in sustaining the growth and invasion of PC cells (24). By contrast, subsequent studies reported that inhibition of AR in murine CAFs increases the expression of stemness markers in co-cultured PC cells. This effect was attributed to the release of interferon-γ (IFN-γ) and macrophage colony stimulating factor (M-CSF) by AR-depleted murine CAFs (25). Consistent with a protective role for stromal AR, it has been reported that expression of the receptor reduces the release of CCL2 and CXCL8 by CAFs, thereby inhibiting PC cell motility (89). These findings further corroborate the idea that PC progression might be paradoxically fostered by ADT or AR blocking therapies. Additional findings have supported an inverse correlation between stromal AR and disease’s malignancy. Immunohistochemistry (IHC) analysis has shown, indeed, that expression of AR is more abundant in non-malignant stroma, as compared with PC-related stroma, further suggesting a protective and antioncogenic role for stromal AR (90–92). In contrast with these findings, our recent study in short-term primary culture of CAFs derived from PC specimens has revealed low, but appreciable AR levels in almost all the CAFs analyzed, even in about 30% of CAFs from PC patients at high Gleason’s score (29). The conflicting findings so far reported might be explained by different considerations. Firstly, the IHC approach for detection of sex steroid receptors often exhibits pitfalls because of the type of cell permeabilization or the primary antibody used (93). Again, AR might be lost in stromal cells as a consequence of CAF selection and/or cell culture manipulation. Additionally, the receptor might undergo degradation as a consequence of ubiquitin-proteasome pathway activation (94) or epigenetic changes (95). Whatever the case, many studies support an oncogenic role for stromal AR, which might induce prostatic intraepithelial neoplasia (PIN; 96) or even metastatic events (97). We have consistently reported that stromal AR directs CAFs towards PC epithelial cells. This process requires AR complexation with filamin A (FlnA) and is strongly stimulated by androgens in 2 and 3D cell culture models. As such, a significant increase in PC-derived organoid’s size can be detected (29). A similar process might occur *in vivo* when the local androgen levels increase, as it frequently occurs in PC (98). Nevertheless, the role of other factors, including NGF, cannot be neglected. In such a way, stromal AR might change the TME composition and allow metastatic events. In support of a role for stromal AR in cancer aggressiveness, it has been shown that AR targeting in CAFs reduces skin cancer aggressiveness traits (26) or even inhibit the development of chemo-resistant skin cancers (27). Thus, AR-directed therapies in CAFs might help the therapeutic approach of different cancer types. We designed and successfully used a small modified peptide that perturbs the androgen-induced AR/FlnA complex assembly (53). By this way, the peptide inhibits the androgen-induced invasion of CAFs in 2D models and reduces the overall tumor size in androgen-treated 3D co-culture (29). Our translational findings identify the AR/FlnA complex as a new ‘druggable’ biomarker in prostate CAFs. The strategy we propose might be profitably used for a more efficient PC treatment.

## Concluding Remarks

PC still represents the second leading cause of cancer death in men in Western society. In most cases (~70%), PC has a slow and symptom-free growth, whereas it is more aggressive in the remaining patients. Current therapies for advanced PC remain unsatisfactory and new inhibitors of androgen synthesis and AR activation have been designed to improve patient survival. Despite these therapies, PC often progress towards a metastatic and/or CRPC phenotype. Preclinical and clinical studies currently aim at identifying the molecular basis for PC spreading and drug-resistance. Nevertheless, few biomarkers predictive of metastatic phenotype have as yet been identified and few efficient therapeutic options are available for advanced PC. Recent years, have seen important advances in large scale –omics approaches to identify novel biomarkers of PC malignancy and ameliorate patients’ stratification as well as clinical management of the disease. The combination of next generation sequencing (NGS) approaches with proteomic profiling has revealed important differences between malignant and benign specimens, together with the identification of clinically actionable biomarkers. Additionally, tools for transcriptional expression classification have allowed the PCs characterization based on their pathological features, thereby addressing the patients towards a proper care (99). Several epigenetic modifications have been also identified and linked to altered gene expression, resulting in increased PC risk. Transcriptome-wide association studies confirmed the correlation with the expression of several predicted genes (100). Similar tools have been also applied to prostate CAFs to show that increased expression of ECM remodelling proteins is linked to cancer-supporting properties (101) or that specific epigenetic alterations are correlated to PC malignancy (102). These approaches might further provide important information on PC molecular landscape.

In this review, we aimed to address novel aspects of PC biology, which impinge on the interconnections between AR and other key intracellular signalling regulated by NGF or calcium channels. Communications between these partners seem relevant, since their breakdown might underly PC pathogenesis and progression. Current PC therapies prevalently target proliferative functions of AR and may only be effective within a short time frame. Primary PC show, instead, a marked heterogeneity and tumor cells may rapidly change as a consequence of pressures exerted by CAFs. Thus, further analysis of tumor microenvironment, identification of its molecular signatures, including the AR expression and its main partners, together with in depth study of signals delivered by PC- or CAF-derived exosomes (103), would provide additional information for patient’s stratification, avoiding expensive therapies with considerable side effects. In this context, the synthesis of new biologically active molecules, such as the new calcium-channel antagonists or the small bioavailable peptides, specifically perturbing the extranuclear AR functions in TME or PC cells, should improve the pharmacologic response in patients and ameliorate their quality of life.

## Author Contributions

FL and GC conceptualized the paper and performed the initial and final editing. FL, MD, and AnM conceptualized and arranged the Figure’s images. All the authors contributed to paper writing and revision. All authors contributed to the article and approved the submitted version.

## Funding

This work was supported by Italian Ministry of University and Scientific Research (P.R.I.N. 2017EKMFTN_002 to GC); VALERE Program (Vanvitelli per la Ricerca Program; GoMAGIC to A.M., AdipCARE to GC and DESIRE to PG). Marzia Di Donato is supported by iCURE Project (B21C17000030007- Regione Campania).

## Conflict of Interest

The authors declare that the research was conducted in the absence of any commercial or financial relationships that could be construed as a potential conflict of interest.

## Publisher’s Note

All claims expressed in this article are solely those of the authors and do not necessarily represent those of their affiliated organizations, or those of the publisher, the editors and the reviewers. Any product that may be evaluated in this article, or claim that may be made by its manufacturer, is not guaranteed or endorsed by the publisher.

## Abbreviations

α-SMA, α-smooth muscle actin; ADT, androgen deprivation therapy; AR, androgen receptor; B2R, bradykinin 2 receptor; BDNF, brain-derived neurotrophic factor; BPH, benign prostatic hyperplasia; CAF, cancer associated fibroblast; CHRM4, cholinergic receptor muscarinic 4; CRPC, castration resistant prostate cancer; CTD, C-terminal domain; EMT, epithelial-to-mesenchymal transition; FAP, fibroblast activating protein; FlnA, filamin A; HER3, Receptor tyrosine-protein kinase erbB-3; HIF-1α, hypoxia-induced factor 1 α; IFN-γ, interferon-γ; IHC, immunohistochemistry; M-CSF, macrophage colony stimulating factor; MAPK, mitogen-activated protein kinase; MHR, melastatin homology region; MMP, matrix metalloproteinase; NEPC, neuroendocrine prostate cancer; NGF, nerve growth factor; NGFR, nerve growth factor receptor; NGS, next generation sequencing; NRG1, neuregulin-1; NT, neurotrophin; PC, prostate cancer; PGC1α, peroxisome proliferator-activated receptor gamma coactivator 1-α; PI3-K, phosphatidylinositol 3-kinase; PIN, prostatic intraepithelial neoplasia; PLCγ, phospholipase C γ; PSA, prostate specific antigen; RTK, receptor tyrosine-kinase; SIRT1, sirtuin 1; TGF-β, transforming growth factor-β; TMD, transmembrane domain; TME, tumor microenvironment; TrkA/B/C, tropomyosin receptor kinase A/B/C; TRPM8, transient receptor potential melastatin-8; VEGF-A, vascular endothelial growth factor-A.
